# The effect of green tea (*Camellia sinensis*) on lipid profiles and renal function in people with type 2 diabetes and nephropathy: a randomized controlled clinical trial

**DOI:** 10.3389/fnut.2023.1253275

**Published:** 2023-12-14

**Authors:** Zeinab Yazdanpanah, Amin Salehi-Abargouei, Zohre Mozaffari, Roya Hemayati

**Affiliations:** ^1^Research Center for Food Hygiene and Safety, School of Public Health, Shahid Sadoughi University of Medical Sciences, Yazd, Iran; ^2^Department of Nutrition, School of Public Health, Shahid Sadoughi University of Medical Sciences, Yazd, Iran; ^3^Yazd Cardiovascular Research Center, Non-communicable Diseases Research Institute, Shahid Sadoughi University of Medical Sciences, Yazd, Iran; ^4^Department of Internal Medicine, Shahid Sadoughi University of Medical Sciences, Yazd, Iran; ^5^Diabetes Research Center, Shahid Sadoughi University of Medical Sciences, Yazd, Iran

**Keywords:** diabetic nephropathy, green tea, lipid profiles, glycated hemoglobin A1c, kidney function

## Abstract

**Introduction:**

Diabetic nephropathy is one of the most important microvascular complications of diabetes. Despite the modern treatments, herbs or medicinal plants have gained wide attention. One of these herbs is green tea (*Camellia sinensis*), which may have an impact on renal function, lipid profiles, and HbA1c. However, the evidence for this is unclear and limited. The present study aimed to evaluate the effect of different doses of green tea on these parameters in type 2 diabetes patients (T2DM) with nephropathy.

**Methods:**

Sixty-six individuals with T2DM nephropathy (aged 30–70 years) were randomly assigned to receive three cups of green tea/day (*n* = 22), two cups of green tea/day (*n* = 22), and the control group (*n* = 22) for 12 weeks. Lipid profiles, glycated hemoglobin A1c (HbA1c), and renal markers were measured before and after intervention. Data were analyzed using SPSS software version 23. One-way analysis of variance (ANOVA), least significant difference (LSD) *post hoc*, and analysis of covariance were used to compare quantitative variables.

**Results:**

In total, 64 participants completed the study. Consuming three cups of infusion green tea per day (7.5 gr) led to a significant reduction in serum levels of total cholesterol (*p* = 0.009) and HbA1c (*p* = 0.006) and increased in high-density lipoprotein cholesterol (HDL-C) (*p* = 0.02) compared with the control group who did not drink green tea. However, no significant differences were observed for other variables.

**Conclusion:**

In general, it was found that drinking three cups of green tea infusion (7.5 gr) per day produced beneficial effects on some lipid profiles and HbA1c without any adverse effects on renal function in patients with T2DM nephropathy. More studies are needed to fully elucidate these findings.

**Clinical trial registration:**

Iranian Registry of Clinical Trials (www.irct.ir) under registry number: IRCT2014020114538N2.

## 1 Introduction

Type 2 diabetes mellitus (T2DM) is now one of the most common metabolic disorders worldwide. This disease is accompanied by a wide range of micro- and macrovascular complications. Diabetic nephropathy (DN) is among the main microvascular complications of diabetes and a major cause of end-stage renal disease (ESRD) (1). DN occurs in approximately 40% of individuals with diabetes (2). It is characterized by abnormal levels of albumin in the urine in the absence of other renal diseases. There are two stages of diabetic nephropathy: microalbuminuria (30–300 mg/24-h urine), and macroalbuminuria or proteinuria (> 300 mg/24-h urine) (3). This disorder is greatly associated with cardiovascular morbidity/mortality and poor quality of life. The specific pathology of DN has remained unclear; however, several mechanisms were determined to participate in its initiation and progression, such as long-term hypertension and hyperglycemia (4), enhanced oxidative stress, inflammatory factors, proteinuria, dyslipidemia, due to genetic susceptibility and dietary factors (5–7).

Currently, available strategies to treat diabetes include oral hypoglycaemic agents and insulin. Despite modern treatments, attention to complementary and alternative approaches such as traditional plant therapies have enhanced in recent decades. The anti-diabetic nephropathy effect of some medicinal plants were also examined and confirmed (8).

Green tea prepared from the leaves of *Camellia sinensis* is one of the traditional medicinal herbs in Asia which is widely consumed worldwide. The tea is high in polyphenolic flavonoids (catechin), quercetin, caffeine, thearubigins, tannins, theaflavins, chlorogenic acid, and other important components (9). Epicatechin (EC), epicatechin-3-gallate (ECG), epigallocatechin (EGC), and epigallocatechin-3-gallate (EGCG) are the most common catechins detected in green tea which were shown to have beneficial effects (10). Phytochemicals and antioxidants in green tea might be effective in managing glucose homeostasis via suppressing hyperglycaemia, preventing kidney glycogen accumulation, protecting kidney function improving glomerular filtration, and reducing oxidative damage and inflammatory reaction via regulating the activity of 5-lipoxygenase, reducing reactive oxygen species (ROS), inhibiting the generation of superoxide radicals and cutting off inflammatory pathways (11).

Recent studies revealed that green tea (*Camellia sinensis*) and its phytochemicals might exert beneficial effects on body weight, blood glucose control and lipid profiles, blood pressure and antioxidant status (12, 13). The positive influence of green tea on renal outcomes were revealed in some animal models of diabetic nephropathy (14). Some previous randomized controlled trials showed no significant effect of green tea polyphenols and extract treatment on glycated hemoglobin A1c (HbA1c) and renal function indicators (15, 16), except albuminuria (16). In a double-blind clinical trial with a 12-week intervention period on women with central obesity, high-dose green tea extract significantly decreased creatinine (Cr) (17). Although a number of trials have examined the effect of green tea on blood glucose markers, data on its effect on renal function and lipid profiles remains controversial and scarce. Moreover, no study has compared the effect of different doses of green tea on type 2 diabetes with nephropathy. Therefore, the present clinical trial was carried out to investigate the effect of different doses of green tea leaves on lipid profiles, glycemic and renal function markers in T2DM nephropathy patients.

## 2 Materials and methods

### 2.1 Study design and participants

The present study was reported based on Consolidated Standards of Reporting Trials (CONSORT) guidelines (18), and was conducted in complete accordance with the Helsinki Declaration (19). This study was approved by the Medical Ethics Committee of Shahid Sadoughi University of Medical Sciences (ethics code: 17/1/141439) and also registered in the Iranian Registry of Clinical Trials (www.irct.ir) under registry number IRCT2014020114538N2. After elaborating on the aim and procedure of this study, informed written consent was signed by all participants.

The present parallel-group randomized controlled trial was conducted on individuals with T2DM and nephropathy in the Diabetes Research Center and related Clinics, Shahid Sadoughi University of Medical Sciences, Yazd, Iran. Participants were recruited according to the eligibility criteria and allocated to three groups using computer-generated random number tables: control group with no treatment (G1, *n* = 22), intervention group with drinking two cups containing 2 g green tea and 200 ml of boiled water (4 g and 400 ml of boiled water) per day (G2, *n* = 22) and intervention group with drinking three cups (7.5 g/750 ml of boiled water) of green tea/day (G3, *n* = 22). Researchers and intervention participants were unaware of the content of the packed sachets (packed and labeled the containers as A or B by another person) until intervention and analysis completion. Furthermore, the biochemical laboratory and the statistician did not know about the allocation either, therefore yielding a double-blind controlled study. The allocation was performed by a person who was unaware of the study protocol. Participants with type 2 diabetes and nephropathy (micro- or macro-albuminuria) aged 30–70 years, with glycated hemoglobin (HbA1c) less than 10%. Participants were excluded if the following criteria were met: having a history of other medical conditions (cancer, liver failure, psychological disorders, gastrointestinal ulcers, and cardiovascular diseases), being on insulin therapy, with hypertension (> 150/90 mm Hg), glomerular filtration rate (GFR) 20 mL/min/1.73 m^2^ or lower, consuming < 70% of close-packed and treating with angiotensin-converting-enzyme (ACE) inhibitors and/or angiotensin receptor blockers (ARBs) for less than 12 months. Subjects who changed their physical activity and routine treatment (dosage and type of medications) were excluded. Pregnant or lactating women, patients who followed a specific diet, or drank more than two cups of black tea/day, and patients who consumed antioxidant supplements were also excluded. Participants in G2 consumed 2 ml × 200 ml of green tea per day (2 g green tea brewed in 200 ml boiling water for 6 min at any time) before lunch and dinner meals for 12 weeks and in G3 were instructed to brew 3 g × 2.5 g green tea in 250 ml of boiled water and drank the tea before main meals (breakfast, lunch, and dinner) the afore-mentioned duration.

### 2.2 Anthropometric and blood pressure measures

The body weight of subjects was measured with a digital scale (Seca, Hamburg, Germany) to the nearest 0.1 kg, with light clothing, bare feet, and after voiding of the bladder. Height measurement was performed using a wall-mounted stadiometer (Seca, Hamburg, Germany) with an accuracy of 0.5 cm. For calculating BMI, the following equation was used: weight (kg)/height^2^ (m^2^). All measurements were done in the morning after the participants had been fasting for at least 8 h. The systolic/diastolic blood pressure (SBP/DBP) was measured while resting in a sitting position for 5-min, by a barometer (Riester, model: diplomat-parameter).

### 2.3 Biochemical analysis

An overnight fasting venous blood sample (10 mL) was taken from each participant at baseline and after 12 weeks of intervention. A calibrated container also collected twenty-four-hour urine samples. The amounts of blood urea nitrogen (BUN), 24-h urine protein (pyrogallol method), and Cr were measured by a commercial kit [Biosystems, Barcelona, Spain; inter− and intra−assay coefficients of variations (CVs) were less than 2.4, 3.2, and 3.9%, respectively]. Written instructions were presented to optimize the accuracy of the 24-h urine collection, and participants reported it; incomplete samples were discarded. The concentration of total cholesterol (TC), high-density lipoprotein cholesterol (HDL-C), and serum triglyceride (TG) were determined using automated enzymatic methods (auto−analyzer machine; Biosystems BA400, Barcelona, Spain). Inter− and intra−assay CVs were 0.93 and 0.62% for total cholesterol, 1.8 and 0.81% for HDL−C, and 1.6 and 1.47% for triglyceride, respectively. Serum low-density lipoprotein (LDL-C) cholesterol was computed using the Friedewald equation (20). Glycosylated hemoglobin (HbA1c) level was measured by high−pressure liquid chromatography method (inter− and intra−assay CVs were less than 2.6%). All biochemical assays for lipid profiles were performed with commercial kits (Pars Azmoon, Tehran, Iran).

### 2.4 Food recall and physical activity

Participants were asked to recall all foods and beverages consumed the previous day when they went to the research center for visiting. Total energy and nutrient intakes were calculated using Nutritionist 4 software (San Bruno, CA, USA) modified for Iranian foods. The International Physical Activity Questionnaire (IPAQ) was completed at the beginning, and end of the intervention to assess the level of individuals’ physical activity (21). All participants were requested to maintain their habitual physical activity, and dietary habits. To evaluate compliance, patients were visited four times (baseline, and weeks 4, 8, and 12) to provide 24-h dietary recalls, and to bring back the remaining packed sachets. A trained dietitian completed questionnaires.

### 2.5 Sample size calculation, dose, and type of green tea leaves

Based on the primitive information obtained from the research of Suliburska et al. (22) for LDL-C (power of 80%, and a confidence interval of 95%), by the following formula: *n* = [(Z_α__/2_ + Z_β_)^2^ × {2(ó)^2^}]/(μ1–μ2)^2^ (23), the sample size was estimated to be 20 participants per group. Finally, according to the probable missing, 22 subjects were considered in each group. Moreover, the green tea dosage was selected according to some previous studies (24–26), and the brewing method of a cup of green tea (without sugar or sweetener) was derived from the study done by Toolsee et al. (26). Green tea leaves were purchased from a local store, and then the species was recognized, and authenticated in the laboratory of the School of Pharmacy, and Pharmaceutical Sciences Shahid Sadoughi University of Medical Sciences and voucher number of the specimen was SSU0094.

### 2.6 Statistical analysis

All statistical analyses were performed using statistical packages for social sciences (SPSS, Chicago, IL, USA, version 23.0). Data are presented as mean ± standard deviation (SD) or standard error (SE). One−sample Kolmogorov–Smirnov test was used to test the normal distribution of the data, and in case of a skewed distribution, logarithmic transformation was applied. Within−group differences were compared using paired *t*-test in each group. To compare the change values among the intervention groups, one-way analysis of variance (ANOVA) and least significant difference (LSD) *post-hoc* tests were utilized. Age, gender, and baseline values were adjusted as covariates in the analysis of covariance (ANCOVA). *P*−values less than 0.05 were considered statistically significant in all analyses.

## 3 Results

**FIGURE 1:**
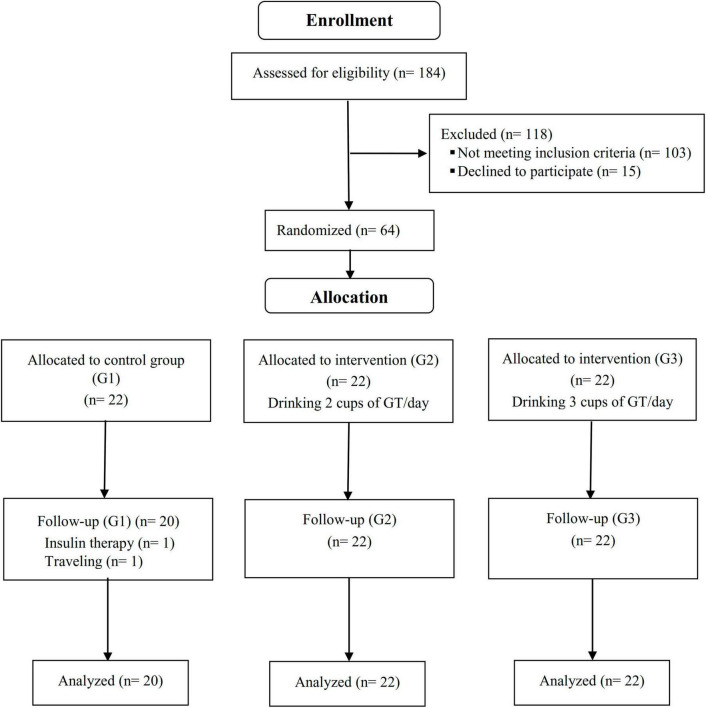
Summary of participants’ flow diagram.

A total of 66 patients (22 subjects in each group) who met inclusion criteria were enrolled in the study. Two of the participants in the control group left the study due to insulin therapy and migration; therefore, 64 participants completed a 12-week period (Figure 1). The mean age and BMI of the participants were 56.46 ± 8.32 (years) and 28.40 ± 3.91 (kg/m2), respectively. Based on the residual green tea in the packed sachets, participants had high adherence (compliance rate was 95.22, 94.72, and 94.27% in G1, G2, and G3 groups, respectively), and no serious adverse events were associated with drinking green tea.

Table 1 indicates the general characteristics of the three groups at the baseline of the study. As observed, none of the characteristics were significantly different among the study groups. Based on 24-h dietary recall, dietary energy and nutrient intake remained stable during the study period, and no significant difference in dietary nutrients was observed between study groups. Both between- and within-group analyses also indicated no significant difference in the physical activity levels (Table 2).

**TABLE 1:** Baseline characteristics of the study participants.

Variables	Control group (*n* = 22)	2 cups GT/day (*n* = 22)	3 cups GT/day (*n* = 22)	*p*-value^a^
Age (y)	58.20 ± 6.10	57.05 ± 8.09	54.15 ± 10.14	0.28
Weight (kg)	72.82 ± 12.36	80.47 ± 13.20	73.72 ± 8.40	0.08
Height (cm)	162.72 ± 10.00	164.95 ± 12.47	163.85 ± 9.23	0.8
BMI (kg/m2)	27.58 ± 4.51	29.49 ± 3.11	27.55 ± 3.14	0.16
Gender (female), *n* (%)	9 (40.90)	9 (40.90)	11 (55.00)	0.76
Menopause status, *n* (%)	5 (22.70)	6 (27.30)	5 (22.70)	0.92
Duration of diabetes (y)	15.40 ± 5.66	14.75 ± 7.84	14.58 ± 7.14	0.91
Smokers (%)	2 (9.10)	4 (18.20)	2 (9.10)	0.56
Compliance (%)	95.22 ± 3.58	94.72 ± 3.01	94.27 ± 3.61	0.66
**Antilipidemic medication, *n* (%)**				
Statins	5 (22.70)	8 (36.40)	6 (27.30)	0.59
Gemfibrozil	7 (31.80)	5 (22.70)	3 (13.60)	0.35
**Antidiabetic medication, *n* (%)**				
Glibenclamide	12 (54.50)	7 (31.80)	9 (40.90)	0.30
Gliclazide	6 (27.30)	9 (4.90)	10 (45.50)	0.43
Metformin	22 (100)	22 (100)	22 (100)	
**Serum factors (units)**				
TC (mg/dL)	167.42 ± 48.54	161.70 ± 40.58	170.55 ± 36.19	0.79
LDL−C (mg/dL)	101.02 ± 33.51	90.74 ± 31.46	94.48 ± 30.16	0.58
HDL−C (mg/dL)	43.99 ± 14.51	43.45 ± 11.86	42.90 ± 6.60	0.95
TG (mg/dL)	192.22 ± 74.67	176.55 ± 75.83	204.72 ± 97.41	0.56
FBS (mg/dL)	143.00 ± 23.82	139.65 ± 27.37	152.00 ± 25.11	0.29
HbA1c (%)	7.43 ± 0.90	7.66 ± 0.96	7.65 ± 0.99	0.71
SBP (mm Hg)	130.43 ± 1.45	120.93 ± 1.54	120.75 ± 1.07	0.24
DBP (mm Hg)	80.65 ± 0.76	80.11 ± 0.84	80.38 ± 0.73	0.07
24-h urine protein (mg)	883.82 ± 261.07	930.90 ± 255.36	795.17 ± 270.05	0.26
BUN (mg/dl)	39.90 ± 10.82	41.20 ± 19.90	38.15 ± 10.57	0.91
Creatinine (mg/dl)	1.16 ± 0.33	1.21 ± 0.39	1.13 ± 0.32	0.86

^*a*^Differences between groups were evaluated using one-way ANOVA test for continuous variables and chi−square test for categorical variables. Data are expressed as mean ± standard deviation (SD) or number (percentage) of patients. BMI, body mass index; ACE, angiotensin-converting-enzyme; ARBs, inhibitors and/or angiotensin receptor blockers; TC, total cholesterol; LDL-C, low-density lipoprotein cholesterol; HDL-C, high-density lipoprotein cholesterol; TG, triglycerides; HbA1c, glycated hemoglobin; BUN, blood urea nitrogen.

**TABLE 2:** Dietary intake and physical activity of participants.

Variable	Control group (*n* = 22)	2 cups GT/day (*n* = 22)	3 cups GT/day (*n* = 22)	*p*-value^b^
**Energy (Kcal)**				
Before	1,927.65 ± 187.56	1,993.45 ± 293.27	1,860.60 ± 223.66	0.22
After	1,901.15 ± 172.04	1,947.85 ± 238.83	1,826.80 ± 203.37	0.18
*P* ^a^	0.18	0.14	0.1	
**Carbohydrate (% of energy)**				
Before	54.20 ± 3.10	53.75 ± 5.34	55.40 ± 3.58	0.43
After	55.25 ± 2.19	54.90 ± 1.68	54.50 ± 1.31	0.41
*P* ^a^	0.15	0.32	0.32	
**Protein (% of energy)**				
Before	13.95 ± 1.66	14.40 ± 2.41	13.90 ± 2.33	0.72
After	14.12 ± 1.07	14.00 ± 0.91	14.50 ± 1.45	0.38
*P* ^a^	0.68	0.49	0.37	
**Fat (% of energy)**				
Before	31.60 ± 3.58	31.40 ± 5.68	30.55 ± 3.96	0.73
After	30.60 ± 2.03	31.05 ± 1.60	30.70 ± 1.49	0.68
*P* ^a^	0.19	0.78	0.87	
**PA (MET−h/week)**				
Before	23.59 ± 16.90	22.22 ± 12.69	22.93 ± 14.55	0.86
After	24.50 ± 17.48	21.79 ± 13.63	23.60 ± 16.69	0.95
*P* ^a^	0.11	0.45	0.51	

^*a*^Within group comparison with paired samples *t*-test.

^*b*^Between groups comparison with one-way ANOVA test. Data are expressed as mean ± standard deviation (SD). PA, physical activity.

Within- and between-group comparisons of lipid profiles, HbA1c, blood pressure, and renal markers are provided in Table 3. The analysis revealed that TC was significantly decreased in the G3 group after 12 weeks of intervention (*p* = 0.01). The comparison of groups who drank three cups of green tea/day and those who did not consume green tea (G1) showed a significant effect of green tea infusion (7.5 g/day) on serum TC levels (−25.00 ± 9.27 mg/dL, *p* = 0.009). In all groups, no significant changes were observed in LDL−C, BUN, and Cr after 12 weeks’ intervention (*p* > 0.05), and also we found no significant differences between the groups. As observed in Table 3, there was a marginal difference among the mean changes of HDL−C in three groups in crude and adjusted models (*p* = 0.06), whereas, according to the LSD *post-hoc* test, the value of HDL−C was increased (6.64 ± 0.02 mg/dL, *p* = 0.02) in the group drinking three cups of green tea compared with the control group. Moreover, a marked difference in HbA1c changes was observed among the three groups in unadjusted (*p* = 0.03) and adjusted (*p* = 0.02) models. LSD *post-hoc* tests showed that the levels of HbA1c reduced in the group receiving 7.5 gr of green tea powder per day compared with the control group (−0.58 ± 0.2%, *p* = 0.006). The analysis revealed that TG levels were not significantly reduced compared with baseline levels in all study groups (*p* > 0.05). Furthermore, a comparison of changes in TG concentrations among three groups revealed no significant difference in crude (*p* = 0.84) and adjusted (*p* = 0.97) models. Besides, this study’s results did not find any significant effect of this treatment on blood pressure among the groups. After adjusting, results remained non-significant for the levels of SBP and DBP (*p* > 0.05). After 12 weeks of study, 24-h urine protein decreased in all groups. However, there was no significant difference among the groups in the levels of change (*p* > 0.05) (Table 3).

**TABLE 3:** Effects of green tea supplementation on serum lipid profile, HbA1c and renal markers.

Variables	Control (*n* = 20)	2 cups GT/day (*n* = 22)	3 cups GT/day (*n* = 22)	*p*-value^a^
**TC (mg/dL)**				
After	172.72 ± 8.64	169.20 ± 10.59	149.35 ± 5.32	0.11
Change^b^	5.30 ± 7.96	7.50 ± 5.43	−21.20 ± 8.02	0.01
Adjusted change	5.51 ± 6.46	5.56 ± 6.42	−19.48 ± 6.51*^†^	0.01^c^
*P* ^d^	0.50	0.18	0.01	
**LDL−C (mg/dL)**				
After	94.66 ± 8.63	88.68 ± 7.96	80.51 ± 4.63	0.39
Change^b^	−6.36 ± 6.67	−2.05 ± 4.80	−13.97 ± 6.26	0.40
Adjusted change	−4.67 ± 5.76	−3.60 ± 5.67	−14.10 ± 5.75	0.37
*P* ^d^	0.35	0.67	0.06	
**HDL-C (mg/dL)**				
After	40.17 ± 2.17	43.95 ± 2.88	45.95 ± 2.50	0.27
Change^b^	−3.81 ± 2.48	0.50 ± 1.42	3.05 ± 2.15	0.06
Adjusted change	−3.65 ± 1.88	0.57 ± 1.87	2.81 ± 1.90*	0.06
*P* ^d^	0.14	0.72	0.17	
**TG (mg/dL)**				
After	184.87 ± 14.47	172.00 ± 15.52	196.55 ± 19.90	0.58
Change^b^	−7.35 ± 4.68	−4.55 ± 5.03	−8.17 ± 4.28	0.84
Adjusted change	−7.41 ± 4.14	−6.32 ± 4.13	−6.33 ± 4.18	0.97
*P* ^d^	0.13	0.37	0.07	
**HbA1c (%)**				
After	7.78 ± 0.19	7.51 ± 0.13	7.32 ± 0.18	0.16
Change^b^	0.35 ± 0.13	−0.14 ± 0.21	−0.33 ± 0.17	0.03
Adjusted change	0.27 ± 0.14	−0.10 ± 0.14	−0.31 ± 0.14*	0.02
*P* ^d^	0.02	0.50	0.07	
**SBP (mm Hg)**				
After	110.86 ± 0.21	110.59 ± 0.22	110.75 ± 0.17	0.65
Change^b^	−1.56 ± 0.21	−1.34 ± 0.20	−1.00 ± 0.17	0.14
Adjusted change	−1.37 ± 0.15	−1.48 ± 0.15	−1.14 ± 0.15	0.31
*P* ^d^	< 0.001	< 0.001	< 0.001	
**DBP (mm Hg)**				
After	70.61 ± 0.15	70.52 ± 0.12	70.52 ± 0.14	0.87
Change^b^	−1.04 ± 0.14	−0.59 ± 0.13	−0.86 ± 0.17	0.11
Adjusted change	−0.87 ± 0.13	−0.73 ± 0.13	−0.89 ± 0.13	0.66
*P* ^d^	< 0.001	< 0.001	< 0.001	
**24-h urine protein (mg)**				
After	771.80 ± 60.16	836.30 ± 52.62	728.15 ± 55.92	0.39
Change^b^	−112.02 ± 21.18	−94.60 ± 18.70	−67.02 ± 12.79	0.21
Adjusted change	−107.70 ± 17.05	−88.50 ± 17.08	−77.44 ± 17.38	0.46^c^
*P* ^d^	< 0.001	< 0.001	< 0.001	
**BUN (mg/dl)^††^**				
After	35.72 ± 2.33	40.24 ± 4.18	37.70 ± 2.22	0.64
Change^b^	−4.17 ± 2.11	−0.96 ± 0.80	−0.45 ± 1.14	0.45
Adjusted change	−4.11 ± 1.42	−0.72 ± 1.41	−0.74 ± 1.43	0.16
*P* ^d^	0.06	0.24	0.69	
**Creatinine (mg/dl)^††^**				
After	1.26 ± 0.09	1.22 ± 0.07	1.14 ± 0.07	0.68
Change^b^	0.10 ± 0.06	0.008 ± 0.03	0.006 ± 0.03	0.61
Adjusted change	0.10 ± 0.04	0.015 ± 0.04	−0.002 ± 0.04	0.23
*P* ^d^	0.17	0.51	0.76	

^*a*^Between groups comparison using one-way ANOVA test.

^*b*^Crude change from baseline without adjusting.

^*c*^Between groups comparison using analysis of covariance (ANCOVA), adjusted for age, sex and baseline values as the covariates.

^*d*^Within group comparison using paired *t*-test.

*p < 0.05 versus control group.

^†^*p* < 0.05 versus G2 group.

^††^These values were log transformed to normalize the distributions. Values are reported as mean ± standard error (SE). TC, total cholesterol; LDL-C, low-density lipoprotein cholesterol; HDL-C, high-density lipoprotein cholesterol; TG, triglycerides; HbA1c, glycated hemoglobin; SBP, systolic blood pressure; DBP, diastolic blood pressure; BUN, blood urea nitrogen.

## 4 Discussion

This randomized, controlled trial aimed to determine the effect of 12−week green tea infusion on renal function, lipid profiles, and glycemic control among patients with T2DM nephropathy. The results of this research showed that drinking three cups of green tea/day (7.5 g) improved serum TC, HDL-C, and HbA1c levels.

To date, several trials have investigated the hypolipidemic effects of green tea in humans. In contrast to the present findings, some clinical experiments revealed no significant effects of green tea on TC and HDL-C levels (24, 27, 28). However, a number of trials found its beneficial effect on lipid metabolism (22, 29, 30). Besides, in a recent meta-analysis conducted by Li et al. (data from 16 trials) (12) on the effect of green tea extract supplementation in obesity with metabolic syndrome, was reported that supplementation with this extract led to a marked increase in HDL-C and decrease in LDL-C, whereas this tea did not improve other lipid profiles. In another meta-analysis which aimed to explore the effects of green tea on lipid parameters in overweight or obese (twenty-one articles), it was shown that green tea had an influential reduction on TC and LDL-C levels in overweight or obese individuals without affecting other lipid parameter (31). No significant changes in LDL-C and TG levels after consuming green tea in this study are consistent with findings of Senger et al. research (28) that was done in elderly with metabolic syndrome who used 3 g/day of green tea for 60 days. Similar results have also been reported in other studies (17, 24, 32); on the other hand, these findings are not in agreement with some results (13, 22, 33). As it was mentioned in both meta-analysis (12, 31), there were discrepancies in the type (pure EGCG to green tea without purity) and dose of the prescribed green tea and the ages and health conditions of study participants. Hence, it seems that the differences in results might be related somewhat to these cases. Although the exact hypolipidemic mechanisms of green tea are not clear, some studies reported that EGCG is the major bioactive catechin responsible for these effects. EGCG suppresses cholesterol biosynthesis with the inhibition of the activity of 3-hydroxy-3-methyl-glutaryl-CoA reductase (HMGR) by competitively binding to the cofactor site of the reductase and interferes with lipid absorption (34, 35). Moreover, following green tea supplementation the fecal excretion of cholesterol increases, and then the activity of cholesterol 7α-hydroxylase stimulates (36).

Green tea also has the potential to improve HbA1c. In line with the findings of current study, several trials indicated significant improvement in HbA1c with green tea administration (17, 37), whereas this positive effect has not been observed by others (15, 16, 26). A recent meta-analysis study including 27 trials, have revealed that green tea consumption did not significantly affect HbA1c levels (38). Contrary to this result, another meta−analysis with seventeen trials found a significant effect of green tea administration on HbA1c (39). Most of the included studies in both meta−analysis were conducted on subjects with overweight/obese; however, our study investigated the role of green tea on T2DM with nephropathy. Some animal evidence indicated the possibility that tea catechins inhibit glucose production by gluconeogenesis (activating 5’-AMP-activated protein kinase) and expression of key gluconeogenic genes (40). Moreover, tea catechins decrease carbohydrate absorption from the intestine via sucrose, alpha-glycosidase, and alpha-amylase enzyme inhibition (41). Moreover, these polyphenol compounds are powerful antioxidants which could ameliorate oxidative stress and induce an enhancement of insulin sensitivity and glucose metabolism (42). The presence of caffeine in green tea can be noted as a possible mechanism for glucose control (43).

Our results presented that green tea consumption (7.5 gr/day) did not affect renal factors (24-h urine protein, BUN, and Cr). However, these data indicated that the consumption of green tea (two/three times a day before meals) is not associated with adverse effects on renal function. In a study conducted by Essex et al. (44), serum Cr and urea concentrations were unchanged following the consumption of three cups per day of green tea for a period of 4 weeks. In support of our results, Toolsee et al. (26) did not observe significant changes in albumin, serum creatinine, urinary creatinine, and urea levels after using 3 cups of green tea (2 gr were infused in 200 mL hot water for 6 min) in individuals at risk of diabetes for 14 weeks, as well as several surveys reached these results (13, 15, 27, 44). Borges et al. (16) showed that the use of green tea polyphenols (containing 800 mg of epigallocatechin gallate) for 12 weeks in patients with diabetic nephropathy has beneficial effects on albuminuria. Furthermore, they indicated that decreasing podocyte apoptosis via activation of the WNT (Wingless integrated pathway) pathway has contributed to the antiproteinuric effect. Another study displays a significant effect of high-dose green tea extract on serum creatinine improvement, which is contrary to our findings (17). This discrepancy may be due to some factors like the type of green tea supplement (composition of pure extract or green tea beverages), consumption dosage, the health status of the population, and intervention duration. Although the exact mechanisms are still controversial, some animal studies suggested which catechins could have a protective effect on renal function by decreasing methylglyoxal signaling and free radical production, increasing antioxidant genes, and suppressing pro-inflammatory mediators. It seems that green tea flavonoids reduce ROS by activating PPARγ, enhancing nuclear factor-erythrocyte-associated factor 2 (Nrf2), and regulating Mn superoxide dismutase production (45). Another possible mechanism may be related to the reduction of the permeability of the glomerular filtration membrane, which inhibits thrombosis via regulating arachidonic acid cascade system and improves microsomal phospholipase A2 activity (PLA2) (46). Besides, green tea catechins can be effective in inhibiting advanced glycation end-product formation and inflammatory pathways by metabolite methylglyoxal trapping and result in ameliorating diabetic nephropathy (47). Furthermore, it appears that regulating the activity of lipoxygenase (inhibition of the leukotriene B4 production) and reducing oxidative stress (superoxide radicals, oxidized proteins, and lipid peroxides) as a result of tea polyphenols can be noted as possible mechanisms for diminishing renal oxidative damage and inflammatory reactions (48).

The present study has some strengths that need to be addressed. To the best of our knowledge, this is the first study that explored the effect of two dosages of green tea infusion on serum lipid profiles (TC, LDL-C, HDL-C, and TG) and renal function markers (24-h urine protein, BUN, and creatinine) in individuals with T2DM nephropathy who were on oral antidiabetic drugs. Moreover, dietary intake and physical activity levels were assessed in the study protocol. It was a parallel randomized controlled trial with excellent adherence to recommended treatment and a low rate of dropout during the intervention. Besides, several potential confounders, such as age, baseline values, and sex were measured in this study. Given these strengths, some potential limitations should be considered, including failure to evaluate the composition of polyphenols in green tea leaves and failure to evaluate oxidative stress markers. Caution is advised when interpreting these results because of the small sample size, and further well-designed and large-scale studies are needed to evaluate the effect of different dosages of green tea on the health status of patients with T2DM nephropathy.

## 5 Conclusion

The present investigation provides findings that the consumption of green tea infusion (7.5 gr/day) could be effective in the improvement of serum TC, HDL-C, and HbA1c levels without any adverse effects on renal function in patients with T2DM nephropathy. However, due to the limitations of the current study, large-scale trials with longer periods and different dosages of green tea are required to better understand its impact, safety, and appropriate dosage for long-term use.

## Data availability statement

The original contributions presented in this study are included in this article/supplementary material, further inquiries can be directed to the corresponding author.

## Ethics statement

The studies involving humans were approved by the Medical Ethics Committee of Shahid Sadoughi University of Medical Sciences (ethics code: 17/1/141439) and also registered in the Iranian Registry of Clinical Trials (www.irct.ir) under registry number IRCT2014020114538N2. The studies were conducted in accordance with the local legislation and institutional requirements. The participants provided their written informed consent to participate in this study.

## Author contributions

The study protocol was designed by RH and ZY. Recruitment of the study participants and data collection were carried out by ZY and ZM. AS-A and ZY provided counseling for statistical analysis. ZY wrote the first draft of the manuscript. The manuscript was critically reviewed by AS-A and RH. All authors read and approved the final version of the manuscript.
